# Faith-Based Community Members, Family, and COVID-19: The Role of Family Cohesion, Social Support, and Spiritual Support on Quality of Life, Depression, and COVID-19-Prevention Behaviors

**DOI:** 10.3390/ijerph191912267

**Published:** 2022-09-27

**Authors:** Kevin Bradley Wright, Rochelle Davidson Mhonde

**Affiliations:** 1 Department of Communication, George Mason University, Fairfax, VA 22030, USA; 2 Department of Global and Community Health, George Mason University, Fairfax, VA 22030, USA

**Keywords:** faith-based communities, family cohesion, social support, spiritual support, health outcomes

## Abstract

This study examined relationships between family cohesion, social support/spiritual support, and quality of life and depression among faith-based community members during the 2020 COVID-19 restrictions. Drawing upon the buffering model of social support and family cohesion as theoretical frameworks, the authors examined these factors in a survey of 551 faith-based community members between March 2020 and June 2020. Family cohesion had a direct and indirect effect (mediated by overall social support and spiritual support on quality of life). Moreover, family cohesion only had a direct effect on depression (e.g., not mediated by overall social support or spiritual support). Greater family cohesion and overall social support were predictive of increased COVID-19-prevention behaviors, while spiritual support was predictive of reduced COVID-19-prevention behaviors.

## 1. Introduction

The 2020 COVID-19 pandemic shutdown altered the communication patterns for millions of U.S. families [1,2]. This included having to live in close quarters for an extended period of time in some families and/or not being able to visit some family members due to travel restrictions or to avoid spreading COVID-19 to more vulnerable family members [2]. In some cases, families experienced increased face-to-face interaction while other families were separated and only able to communicate via technology. These changes to family living conditions and family communication patterns likely affected factors such as family cohesion, which has been found to influence family coping, mental health, and quality of life [1,3,4]. However, few studies have examined family cohesion, social support, and health outcomes within the context of a health crisis such as COVID-19 or among members of faith-based communities.

By the end of March 2020, the COVID-19 virus had spread to all U.S. states and many countries around the world. During Spring 2021 when many of the restrictions began to lift, there were over 600,000 deaths due to COVID-19 in the U.S., and most states still had shelter-in-place orders or restrictions to large crowds, including for religious purposes [5]. In addition to the physical health threats posed by COVID-19, mental health issues have been documented as one of the most frequent types of health concern during the COVID-19 pandemic in the U.S. [6]. These include continuous emotional and behavioral difficulties such as depression and anxiety symptoms associated with the pandemic [7,8,9]. The successive waves of the COVID-19 pandemic and the emergence of multiple virus variants significantly impacted the communication patterns within a number of institutions, including families and faith-based communities [10,11]. Moreover, during the 2020 COVID-19 shutdown (and beyond), many people in the U.S. experienced interpersonal conflicts with other family members over mask and vaccine mandates that were often divided over political party and ideological lines (including those associated with certain faith-based communities) that may have influenced perceptions of family cohesion [10,12,13]. In addition, travel restrictions kept some families apart during the height of the pandemic shutdown, and this may have influenced perceptions of family cohesion during this time period. In short, we would expect that perceptions of family cohesion would vary during this time period, with some families experiencing greater cohesion than others.

Furthermore, previous studies have found that religion and spirituality may help many individuals cope during stressful life events, such as the COVID-19 pandemic [11,14,15]. Religion and spirituality have a positive impact on mental health by providing social support and spiritual support to faith-based community members [16,17]. In times of crisis, humans have a tendency to turn to religion for comfort and explanation [11,15]. Within the context of COVID-19, religion and spirituality also appear to have had positive mental health effects on faith-based community members coping with the anxiety caused by the pandemic [11,18,19].

Much of the information about COVID-19 that was circulating on social media during this time period contained misinformation or disinformation about the nature of the COVID-19 virus itself, the efficacy of masks and vaccines, mortality rates, and a host of other COVID-19 related topics [20]. This led to higher levels of uncertainty about the severity and duration of the pandemic and how it might continue to disrupt normal life, which added to people’s anxiety and depression levels [20,21,22]. However, less is known about how faith-based community members’ families and larger social support networks influenced their perceptions of quality of life and depression levels during this period of the COVID-19 pandemic. In addition, little is known about how family cohesion and social support impacted faith-based community members engagement in COVID-19-prevention behaviors.

The current study examines relationships between perceptions of family cohesion, social support/spiritual support, and perceived depression and quality of life among faith-based community members during the 2020 COVID-19 pandemic shutdown in the U.S. Toward that end, the authors draw upon the buffering model of social support and family cohesion as theoretical frameworks. Moreover, we examine previous research on faith-based communities, family cohesion, the influence of social support and spiritual support on mental health outcomes (quality of life and depression). This is followed by a report from a survey of faith-based community members that was conducted between March 2020 and June 2020 (during the beginning of U.S. COVID-19 restrictions).

## 2. Religion, Faith-Based Communities, Social Support, and Mental Health Outcomes

For many Americans, their faith provides the lens through which they approach most matters of importance, and a health crisis on the scale of the COVID-19 pandemic certainly seems tailor-made for turning to religion. Yet, the religiosity of Americans poses a unique challenge during a pandemic such as COVID-19, as the comfort that religion offers may be challenging to obtain due to the many social distancing protocols. Religious worship generally supports close social interaction and social support, which provides many mental and physical health benefits, but it can also contradict infection control measures, such as preventative behaviors in the case of COVID-19 [23,24]. For example, some faith-based communities may have exacerbated stress and anxiety among their members, especially when congregations and religious leaders held ideological positions based on their religious doctrine that discouraged vaccines, masks, or social distancing recommendations (e.g., those communities that continued to hold religious mass gatherings during the COVID-19 shutdown). Additionally, some faith-based communities led members to believe that the efficacy of prayer or belonging to their particular faith community would provide miraculous protection from COVID-19, making masks or social distancing unnecessary [25].

Making the decision to attend religious services versus complying with social distancing was likely not always easy for faith-based community members during the COVID-19 shutdown. Some people had to weigh the value derived from religious social involvement against the uncertainty regarding risk and susceptibility to contracting COVID-19 within this context [26]. A few large religious gatherings during this time period were documented as “super-spreader events” for COVID-19 transmission when social distancing was not practiced [23,24,27]. Furthermore, social distancing may have been especially difficult for religious-minded individuals who preferred to counteract the negative effects of isolation due to COVID-19 by worshiping, seeking support, and communing with other members of their faith-based communities.

## 3. Family Cohesion, Social Support, and Health Outcomes

Communication between family members (including those of faith-based community members) has the potential to facilitate coping with uncertainty in times of crisis, such as the upheaval triggered by the global COVID-19 pandemic [28]. During the 2020 COVID-19 shutdown in the U.S., many families and their family communication patterns were altered by the pandemic [29]. For some families, this meant quarantining under the same roof, which led to family members spending more time with each other compared to pre-pandemic levels of interaction [29]. Other families were geographically separated, and many opted to communicate virtually via social media or cell phone to avoid spreading the virus to older or more vulnerable family members or to avoid traveling long distance during the shutdown [29]. Researchers have concluded that such changes to family interaction likely influenced perceptions of family cohesion, especially during the height of COVID-19 restrictions [10,30].

*Family cohesion* refers to the emotional bonding that family members have toward one another [31], and it depends on how systems balance their separateness versus togetherness [32]. Family cohesion is defined as shared affection, support, helpfulness, and caring among family members [33], and it has been examined in a variety of family communication and health outcome studies [34,35,36]. Many scholars conceptualize that there are varying levels of family cohesion, ranging from disengaged (very low) to enmeshed (very high) families [31,33]. Members in highly cohesive families tend to unite to resolve their problems and typically provide one another greater social support compared to less cohesive families [32]. As a result, members of more cohesive families tend to experience greater perceived social support compared to individuals in less cohesive families [32,37].

Previous studies have suggested that family cohesion during a traumatic event can improve perceived quality of life [28,38], including during the COVID-19 pandemic [39,40,41]. Families that are emotionally closer appear to effectively communicate to cope with stressors and, in turn, improve quality of life [32,42,43]. For example, high family cohesion has been linked to positive, supportive interaction among family members that is positively and linearly related to individual and family functioning as well as health outcomes such as reduced depression and stress [44,45].

Other studies have found that COVID-19 restrictions influenced family social support patterns, with some families benefitting from spending more time with family members, while other families perceived less support from family members [29,46]. Many other families experienced a variety of problems during the pandemic, including conflict, substance abuse, domestic abuse, divorce or separation, and the death of a family member (due to COVID or other causes) [47,48,49,50], which likely influence depression and quality of life. However, less is known about how family cohesion directly and indirectly influences health outcomes such as depression and quality of life through social support behaviors, particularly during an unprecedented event such as COVID-19. Moreover, little is known about how family cohesion is related to COVID-19-prevention behaviors among faith-based community members.

## 4. Buffering Model of Social Support and Types/Sources of Support for Faith-Based Community Members

Another theoretical framework that has been used extensively in the study of social support and mental health outcomes such as depression and quality of life is the buffering model of social support [51]. Cobb (1976) first introduced the concept of the buffering model to explain how social support can protect a person against stress. The buffering model posits that individuals with strong social support systems tend to experience reduced or no negative effects on their health and well-being due to the shielding, or “buffering” effects of social support, including informational support, validation, emotional support, tangible support, and a variety of other forms of support [52,53]. In other words, the social support we receive from our relationships (including family members) appears to help reduce stress levels, which can impact health outcomes such as quality of life and depression.

Spiritual support has been relatively understudied compared to other types of social support (e.g., informational, tangible, emotional support). However, the findings from several previous studies suggest that spiritual support may play an important stress-buffering role for faith-based community members [54,55,56]. Recent scholars have conceptualized spiritual support as perceived support from other faith-based community members and spiritual leaders regarding spiritual matters [57,58]. Seeking spiritual support has been identified as way that many faith-based community members have coped with other major crisis events in the past, such as 11 September [59]. However, it remains unclear the degree to which spiritual support specifically influenced quality of life and depression in combination with other types of social support (e.g., informational, emotional, tangible, etc.) that faith-based community members may have obtain from larger social networks (e.g., family, friends, etc.) during the COVID-19 shutdowns. Additionally, previous studies have not explored how family cohesion may have influenced perceptions of overall social support and spiritual support among faith-based community members during COVID-19, and how this, in turn, influenced depression and quality of life.

## 5. Hypotheses and Research Questions

**Figure 1:**
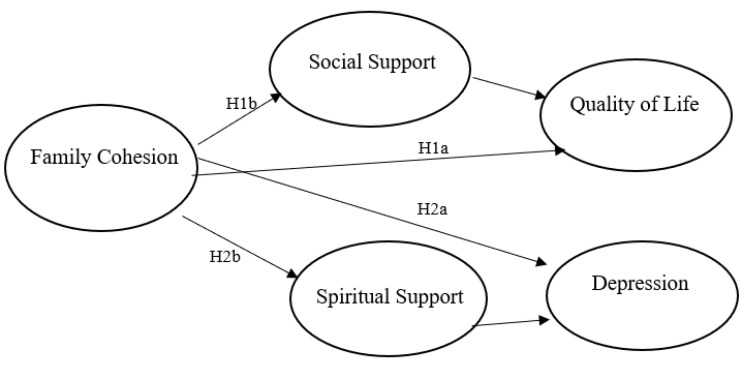
Conceptual model with relationships between key variables (Hypotheses H1a–H2b).

Based on the previous theory and research discussed above, the researchers posed the following research questions. Figure 1 provides a visual representation of the conceptual relationships between variables tested in H1a through H2b.

**Hypothesis** **1a** **(H1a).** Family cohesion will predict higher quality of life among faith-based community members during COVID-19.

**Hypothesis** **1b** **(H1b).** Social support and spiritual support will mediate the relationship between family cohesion and quality of life among faith-based community members during COVID-19.

**Hypothesis** **2a** **(H2a).** Family cohesion will predict lower depression among faith-based community members during COVID-19.

**Hypothesis** **2b** **(H2b).** Social support and spiritual support will mediate the relationship between family cohesion and depression among faith-based community members during COVID-19.

**RQ1.**  Do family cohesion, social support, spiritual support predict COVID-19-prevention behaviors among faith-based community members during COVID-19?

## 6. Methods

### 6.1. Participants

Following IRB approval, participants for the study were recruited via a snowball sample of undergraduate and graduate students at a large, diverse, mid-Atlantic university who self-identified as being a member of a faith-based community. Participants were asked to help the researchers find other members of their faith-based communities and urge them to participate in the survey. In total, N = 551 faith-based community members completed the online survey questionnaire. All respondents to the online survey completed an online informed consent form prior to linking to the survey questionnaire.

Of these individuals, 314 were men, 219 were women, and 1 person identified as non-binary in terms of gender. The average age was 30.55 years (*SD* = 11.17). In terms of race/ethnicity, 234 indicated they were White, 218 participants identified as Asian, 32 said they were Hispanic/Latinx, 30 reported Black/African American, 10 mentioned Middle Eastern, 3 said they were Native American, and 17 mentioned “other”. As far as level of education was concerned, the majority of the sample was well educated, with 229 people indicating they had a bachelor’s degree, followed by 133 with some college, 76 with a master’s degree or equivalent, 60 with a high school diploma, 34 with an associate’s degree, and 2 with a doctoral degree. The majority of the sample (*n* = 117) reported living with a spouse, 104 said they lived with their parents, 97 mentioned living with a spouse and children, 76 said they lived with a roommate or multiple roommates, 62 indicated they lived with their significant other, and 59 people mentioned living alone.

As far as religious affiliation is concerned, the majority of the participants identified as Christians (*n* = 347), followed by Hindu (*n* = 58), Muslim (*n* = 38), Buddhist (*n* = 23), Jewish (*n* = 13), and “Other Religion (*n* = 47)”. Other religions included Bahai, Sikhism, and several types of pagan communities (e.g., Wicca). In addition, the survey questionnaire also asked the degree to which faith-based community members attended religious or spiritual programs within their faith-based community in person using three categories (frequently, sometimes, never). In total, 170 participants indicated that they had frequently attended a face-to-face religious or spiritual program, 196 said they sometimes attended, and 180 mentioned that they had never attended a face-to-face religious or spiritual program.

### 6.2. Measures

**Family cohesion.** The researchers measured family cohesion using the family cohesion scale from the FACES II instrument [30]. This 16-item measure uses a five-point Likert-type scale with the anchors “*strongly disagree* = 1” and “*strongly agree* = 5”. Sample items included “My family members consult other family members on their decisions” and “My family members are supportive of each other during difficult times. We asked participants to focus on their current family (even if they were not currently living with family members) All 16 items were combined to form a single index representing respondents’ level of perceived family cohesion in the past month (*M* = 2.30; *SD* = 0.66; α = 0.92).

**Perceived social support**. Perceived social support was assessed using Schwarzer and Schulz’s (2013) [60] Perceived Support subscale (from the Berlin Social Support Scales—BSSS). This 17-item instrument uses a 5-point Likert-type scale with the anchors (1) *strongly disagree* and *strongly agree* (5), and it includes items such as “There is always someone there for me when I need cheering up”, and “There are people who offer me help when I need it. The items were combined into a single index of perceived support (*M* = 3.74; *SD* = 0.60; α = 0.88).

**Spiritual support.** Spiritual support was measured using the spiritual support subscale of the MOS Social Support Survey [58]. This subscale consists of three 5-point Likert-type scale items with the anchors (1) *strongly disagree* and *strongly agree* (5), including “Whenever I need it, there is someone I can talk to about spiritual matters”, and “Whenever I need help, there is someone who will pray for me (or meditate with me)”. These items were combined to form a single index representing participants’ level of perceived spiritual support, with higher scores representing higher perceived spiritual support (*M* = 3.77; *SD* = 0.94; α = 0.82).

**Depression**. Depression was measured using Noh, Kasper, and Chen’s (1998) adapted version of The Center for Epidemiologic Studies-Depression Scale (CES-D) [61]. The CES-D scale has been widely used in population studies of depression. This 15-item measure uses a 5-point Likert-type scale with the anchors (1) *strongly disagree* and *strongly agree* (5). Sample items include statements such as “I felt depressed”, “I felt inferior to other people”, and “I am pessimistic about the future”. All 15 items were combined to form a single index representing respondents’ level of perceived depression during the past month, with higher scores representing higher perceived depression (*M* = 2.92; *SD* = 0.73; α = 0.87).

**Quality of life (QOL)**. Quality of life (QOL) was measured by a single item, “How has your quality of life been during the past 4 weeks? That is, how have things been going for you?” Participants responded to this question on a 5-point Likert-type scale with the anchors (1) *very bad: could hardly be worse* and (5) *very well: could hardly be better*.

**COVID-19 preventative behaviors.** To measure preventative behaviors related to COVID-19, the current study adapted five items from the Social Risk Factors for COVID-19 Exposure Questionnaire [62]. This scale consists of five 5-point Likert-type items adapted from the Accorsi et al.’s (2020) larger measure with the anchors (1) *never* and (5) *always*. Items included, “I practice social distancing strategies to increase the space between individuals” and “I wear face masks or cloth face coverings when I leave my house”. These items were combined to form a single index representing participants’ level of COVID-19-prevention behavior, with higher scores representing increased prevention behavior (*M* = 2.48; *SD* = 0.21; α = 0.87).

### 6.3. Statistical Analyses

Statistical analyses were conducted using SPSS version 26. Descriptive statistics were conducted to describe sample characteristics. Correlations among variables used in the mediation analysis are listed in Table 1. We used Hayes’s PROCESS macro (Models 4 and 6) version 3.5 for SPSS to estimate mediation effects using bootstrapping [63]. Bootstrap resampling was set to 5000 times for all analyses. PROCESS estimates model parameters via ordinary least squares (OLS) regression and provides path coefficients for direct and indirect effects as unstandardized regression coefficients, as well as bootstrapped 95% confidence intervals (CI). If the CIs do not encompass 0, the indirect effect is significant and supports a mediating effect. A preliminary analysis revealed no statistically significant difference on the key study variables based on religious affiliation, race/ethnicity, income, or education. However, women (*M* = 3.84; *SD* = 0.58) reported significantly higher levels of overall support than men (*M* = 3.65; *SD* = 0.60), *t =* −3.564, *p* < 0.001.

## 7. Results

H1a stated that family cohesion will predict higher quality of life among faith-based community members during COVID-19. A regression analysis found that family cohesion (*M* = 2.30; *SD* = 0.66) had a significant, positive direct effect on quality of life (*M* = 2.39; *SD* = 0.78), *β* = 0.10, SE = 0.05, *t* = 2.250, *p* < 0.05, CI = 0.01, 0.21. To further investigate the relationship between family cohesion and quality of life, we tested the mediating effects of social support and spiritual support (H1b). The mediation analysis revealed that family cohesion (*M* = 2.30; *SD* = 0.66) significantly and positively predicted overall perceived social support (*M* = 3.74; *SD* = 0.60), *β* = 0.22, SE = 0.06, *t* = 5.245, *p* < 0.001, CI = 0.02, 0.28, and a significant positive effect between perceived overall social support (*M* = 3.74; *SD* = 0.60) and quality of life (*M* = 2.39; *SD* = 0.78), *β* = 0.12, SE = 0.07, *t* = 2.256, *p* < 0.05, CI = 0.09, 0.33. There was also a significant and positive indirect effect between family cohesion (*M* = 2.30; *SD* = 0.66) and spiritual support (*M* = 3.77; *SD* = 0.94), *β* = 0.15, SE = 0.06, *t* = 3.441, *p* < 0.001, CI = 0.09, 33, and a significant and positive indirect effect between spiritual support (*M* = 3.77; *SD* = 0.94) and quality of life (*M* = 2.39; *SD* = 0.78), *β* = 0.14, SE = 0.04, *t* = 2.677, *p* < 0.01, CI = 0.03, 0.19 (See Table 2). The total effect of family cohesion on quality of life was positive (*β* = 0.07, *df* = 3516, *F* = 12.588, *p* < 0.001). The model explained 26% of the variance in terms of how family cohesion is related to quality of life. The findings support H1a and H1b.

H2a stated that family cohesion will predict lower depression among faith-based community members during COVID-19. A regression analysis found that family cohesion (*M* = 2.30; *SD* = 0.66) had a significant, direct negative direct effect on depression (*M* = 2.92; *SD* = 0.73), *β* = −0.26, SE = 0.05, *t* = 5.921, *p* < 0.001, CI = −0.19, −0.37. To further investigate the relationship between family cohesion and depression, we tested the mediating effects of social support and spiritual support (H2b). The mediation analysis revealed that family cohesion (*M* = 2.30; *SD* = 0.66) significantly and positively predicted overall perceived social support (*M* = 3.74; *SD* = 0.60), *β* = 0.22, SE = 0.04, *t* = 5.221, *p* < 0.001, CI = 0.13, 0.28. However, there was not a significant negative effect between perceived overall social support (*M* = 3.74; *SD* = 0.60) and depression (*M* = 2.92; *SD* = 0.73), *β* = −0.10, SE = 0.06, *t* = −1.902, *p* = 0.057, CI = −0.004, 24. There was also a significant and positive indirect effect between family cohesion (*M* = 2.30; *SD* = 0.66) and spiritual support (*M* = 3.77; *SD* = 0.94), *β* = 0.15, SE = 0.06, *t* = 3.453, *p* < 0.001, CI = 0.06, 33, but no indirect effect between spiritual support (*M* = 3.77; *SD* = 0.94) and depression (*M* = 2.92; *SD* = 0.73), *β* = −0.03, SE = 0.04, *t* = 0.8905, *p* > 0.05, CI = −0.04, 0.11 (See Table 3). The total effect of family cohesion on depression was negative (*β* = −0.27, *df* = 1515, *F* = 41.306, *p* < 0.001). The model explained 22% of the variance in terms of how family cohesion is related to depression. The findings support H2a but they do not support H2b.

RQ1 asked if family cohesion, overall social support, and spiritual support were predictive of COVID-19-prevention behaviors among faith-based community members during the COVID-19 shutdown. A regression analysis found that higher family cohesion scores (*M* = 2.30; *SD* = 0.66) significantly predicted higher COVID-19-prevention behavior scores (*M* = 2.49; *SD* = 0.21), *β* = 0.259, *t* = 71.45, *p* < 0.001. In addition, a regression analysis found that higher overall social support scores (*M* = 3.74; *SD* = 0.60) predicted increased COVID-19-prevention behaviors (*M* = 2.49; *SD* = 0.21), *β* = 0.20, *t* = 3.959, *p* < 0.001. However, increased spiritual support scores (*M* = 3.77; *SD* = 0.94) predicted lower COVID-19-prevention behaviors (*M* = 2.49; *SD* = 0.21) among faith-based community members, *β* = 0.13, *t* = 3.959, *p* = 008.

## 8. Discussion

The purpose of this study was to examine relationships between family cohesion, social support/spiritual support, and perceived quality of life and depression among faith-based community members during the 2020 COVID-19 pandemic shutdown in the U.S from a buffering model of social support and family cohesion framework. The study findings have a number of theoretical and pragmatic implications for scholars who are interested in the role of family cohesion and social/spiritual support on health outcomes among faith-based community members. This section examines the theoretical and practical implications of the study findings, limitations, and directions for future research.

Consistent with previous empirical studies of family cohesion and the buffering model of social support, the degree to which faith-based community members perceived their family to be cohesive influenced their perceptions of social support and spiritual support, which, in turn, influenced their perceptions of quality of life (QOL) [34,36,55,56]. However, while family cohesion influenced both overall social support and spiritual support for faith-based community members, these sources of social support did not influence depression. Instead, family cohesion had a direct effect on depression. This finding does not support the mediating (i.e., buffering) role of social support. This raises an interesting question regarding how perceptions of family cohesiveness directly influence perceived depression within this population. One explanation may be that perceptions of family cohesiveness may be more enduring than perceived depression, especially during an unprecedented pandemic where perceived depression may have varied depending upon factors such as when participants completed the survey (e.g., earlier in the shutdown vs. later in the shutdown) or due to other life circumstances. Future studies should continue to explore how family cohesion may directly impact health outcomes such as depression and quality of life in a longitudinal study as opposed to using a cross-sectional design as in the current study.

One limitation of the current study is the data were collected at the beginning of the COVID-19 shutdowns in the U.S., which limits the generalizability of the findings. At the time, there were many factors that were not measured in the current study that could account for some of the unexplained variance in the regression models. For example, in the U.S. at this time, there was a high degree of uncertainty, fear, political unrest, worry about job security and family health issues, work–life balance issues in families. These and most likely a host of other factors influenced perceptions of stress, depression, and quality of life. Future studies where longitudinal data are available would benefit in terms of comparing differences in family cohesion, overall support, spiritual support, and their influence on depression and quality of life between the height of COVID-19 restriction and today. Such comparisons might provide insights into how family cohesion, overall support, and spiritual support influence these health outcomes during times of health crisis. This could be useful in terms of developing social support interventions for families during similar health or other disruptive crises in the future.

Another limitation of the study is that the items in the spiritual support scale did not include items dealing with more intrapersonal types of spiritual support (e.g., prayer, etc.). The spiritual support scale used in the current study had participants respond to interpersonal types of spiritual support within one’s faith-based community (e.g., “there is someone who will pray with me”,, etc.). It is unclear whether individuals shifted to more intrapersonal forms of spiritual support during the pandemic that may enhance perceptions of quality of life. However, the focus of the current study was limited to dyadic or interactive forms of spiritual support (e.g., having others pray for your family, etc.) that participants received from their social network (including other members of participants’ faith-based community). Future studies of faith-based communities should continue to differentiate between multiple types/sources of social support in an effort to learn which may be most influential in terms of reducing perceived stress and enhancing quality of life. This finding may be useful to researchers who wish to design a social support-based health intervention among faith-based community members.

The study findings indicated that family cohesion significantly predicted COVID-19-prevention behaviors among faith-based community members during the COVID-19 shutdown. This may have implications for researchers who are interested in the role family cohesion may play in terms of supporting and/or reinforcing prevention behaviors and lifestyle changes with COVID-19 or similar future pandemics among members of faith-based community members. Highly cohesive families provide greater social support and encouragement for behavioral change than families that are less cohesive in previous work [32,43,44]. However, the current study provides evidence that families with greater levels of cohesion may play a role in supporting and encouraging COVID-19-prevention behaviors (and prevention behaviors for future pandemics) for faith-based community members. Future studies should continue to examine the role of family cohesion in efforts to change future pandemic-related behaviors. It was also interesting that overall social support scores predicted increased COVID-19-prevention behaviors. However, one limitation of the study is that overall social support could include family members as well as friends, other faith-based community members, etc. So, family cohesion likely influenced perceptions of support from family members (which would lead to increases in overall perceived support). Future studies would benefit from using social support measures that differentiate between exclusively family support and support from other types of relationships. This could be helpful in terms of understanding the influence of social support on depression and quality of life from specific social network member sources for faith-based community members. Future social support intervention developers should account for the relative influence of multiple sources of perceptions of mental health.

Finally, it was also interesting that increased spiritual support scores predicted lower COVID-19-prevention behaviors among faith-based community members. This might reflect the idea that some individuals who experience high levels of spiritual support might feel that their faith in God or a higher power will protect them from COVID-19 or adverse health outcomes stemming from COVID-19. Another factor to consider is the degree of fatalism in one’s religious/spiritual beliefs. Some faith-based communities may hold beliefs that are more fatalistic in nature than others. In such cases, faith-based community members might hold more fatalistic views about their own health when it comes to COVID-19. For example, this type of fatalistic set of beliefs might lead a person to take fewer precautions against COVID-19 (such as getting vaccinated or wearing a facemask) since he or she may be more likely to believe that God or a higher power will ultimately protect against COVID-19 (regardless of individual prevention behaviors). Future researchers should investigate the role of such religious/spiritual beliefs may influence behaviors when designing disease prevention intervention campaigns (for other pandemics or similar crises that disrupt regular faith-based community activities. Many health campaigns work with faith-based communities as partners in terms of helping to disseminate campaign messages. However, it is important to understand the organizational culture of faith-based communities as well as how cultural influences may influence members’ beliefs and perceptions about a variety of health concerns, including COVID-19.

## Figures and Tables

**Table 1:** Bivariate correlations between cohesion, social support, and health outcomes.

	1	2	3	4	5
Cohesion	1	0.15 **	0.22 **	−0.27 *	−0.14 **
Spiritual Support		1	0.56 **	−0.03	0.21 **
Overall Support			1	−0.12 **	0.21 **
Depression				1	−0.34 **
QOL					1

* *p* < 0.05; ** *p* < 0.01.

**Table 2:** Direct and indirect effects of family cohesion and spiritual support on QOL.

Paths	B	SE	95% CI
*Direct Effect of Family Cohesion*			
Cohesion → QOL	0.10 *	0.05	(0.01, 0.21)
*Mediating Effect of Family Cohesion*			
Cohesion → Spiritual Support	0.15 ***	0.06	(0.09, 33)
*Mediating Effect of Social Support*			
Overall Support → QOL	0.12 *	0.07	(0.09, 0.33)
Spiritual Support → QOL	0.14 **	0.04	(0.03, 0.19)

Note: Cohesion = Family Cohesion, Overall Support = Perceived Overall Social Support, QOL = Quality of Life, * *p* < 0.05, ** *p* < 0.01, *** *p* < 0.001.

**Table 3:** Direct and indirect effects of family cohesion and social support on depression.

Paths	*β*	SE	95% CI
*Direct Effect of Family Cohesion*			
Cohesion → Depression	−0.26 ***	0.05	(−0.19, −0.37)
*Mediating Effect of Family Cohesion*			
Cohesion → Overall Support	0.15 ***	0.04	(0.13, 0.28)
Cohesion → Spiritual Support	0.15 ***	0.06	(0.06, 33)
*Mediating Effects of Social Support*			
Overall Support → Depression	−0.1	0.06	(−0.004, 24)
Spiritual Support → Depression	−0.03	0.04	(−0.04, 0.11)

Note: Cohesion = Family Cohesion, Overall Support = Perceived Overall Social Support, QOL = Quality of Life. *** *p* < 0.001.

## Data Availability

The data presented in this study are available on request from the corresponding author. The data are not publicly available due to privacy issues.
